# *Exocarpium Citri Grandis* ameliorates alcoholic liver disease by modulation of hepatic lipid metabolism and iron homeostasis

**DOI:** 10.1186/s13020-025-01229-4

**Published:** 2025-10-16

**Authors:** Yun-jia Li, Yu-xue Zhang, Shu Xu, Meng-chen Qin, Guang-hui Deng, Jun-jie Li, Xin-feng Shen, Hang Guo, Yu-xin Liao, Chu-ying Zhou, Shao-hui Huang, Ming-hao Liu, Wen-xia Zhao, Lei Gao

**Affiliations:** 1 School of Traditional Chinese Medicine, Southern Medical University, Sha Tai Nan Road No. 1063, Guangzhou, 510515 Guangdong China; 2 The First Affiliated Hospital of Henan University of Traditional Chinese Medicine, 450099 Zhengzhou, China; 3 Cancer Center, Shenzhen Guangming District People’s Hospital, Shenzhen, 518106 Guangdong China; 4 The First Affiliated Hospital/The First Clinical Medicine School of Guangdong Pharmaceutical University, 510224 Guangdong, China

**Keywords:** *Exocarpium Citri Grandis*, Alcoholic liver disease, Zebrafish, Iron metabolism, Lipid peroxidation, RAGE

## Abstract

**Background:**

Alcoholic liver disease (ALD) is a key cause of chronic liver disease worldwide, which progresses to liver cirrhosis and even hepatocellular carcinoma. After years of application and research, the traditional Chinese medicine (TCM) Exocarpium Citri Grandis (ECG) has shown the significant function of lowering lipid and outering the effect of drinking, but the specific mechanism of its action in ALD is not clear.

**Purpose:**

The aim of this study is to investigate the anti-alcohol and lipid-lowering effects of ECG and its underlying pharmacological mechanisms in vitro and in vivo.

**Methods:**

First, this study initiated with a preliminary identification of the active components in the aqueous extract of ECG. Then zebrafish, mice, AML-12 cells and RAW264.7 cells were used as the research object. Serum and hepatic iron concentration were assessed by biochemical assays and iron assay kits. Besides, cells were constructed with the receptor for advanced glycation endproducts (RAGE) overexpression virus for further research.

**Results:**

ECG extract was low-toxicity and effectively alleviated alcohol-induced hepatic steatosis in mice and zebrafish. Besides, ECG extract intervention inhibited hepatic iron overload in ALD through mediating the iron-related pathways. Specifically, ECG reversed the down-regulation of ferroportin1 and up-regulation of hepcidin and ferritin in mice liver induced by alcohol, which subsequently suppressed iron dependent-lipid peroxidation and inflammation. In addition, flavonoids were the main components of ECG and could be combined with RAGE. The study indicated that ECG had a superior therapeutic effect against alcohol-induced liver injury in vivo and in vitro.

**Conclusions:**

The protective effect of ECG might be closely related to the modulation of RAGE-mediated lipid accumulation and iron overload. The evidence provides the therapeutic promise of ECG in ALD.

**Supplementary Information:**

The online version contains supplementary material available at 10.1186/s13020-025-01229-4.

## Introduction

Excessive alcohol consumption poses a significant global health challenge, with alcoholic liver disease (ALD) accounting for over half of liver cirrhosis-related deaths in the United States [1]. Alcohol stands as a prominent trigger and facilitator of liver fibrosis, contributing to the development of various hepatic disorders, the most characteristic of which are steatosis, hepatitis, and cirrhosis [2]. Owing to its high prevalence and substantial economic impact, ALD has garnered increasing attention from the medical community. Nevertheless, long-term abstinence remains the most efficacious approach to alleviate ALD symptoms and potentially reverse liver damage, while liver transplantation serves as the definitive therapy for individuals with alcoholic cirrhosis [3–5]. These realities underscore the pressing need for alternative or adjunctive treatments to address the multifaceted challenges posed by ALD.

The physical examination of patients with ALD only shows mild tender hepatomegaly, which is difficult to be diagnosed clinically. At present, there is no effective regimen for the treatment of ALD in clinical practice, and it is still a major challenge to find safe and effective prevention and treatment methods. Traditional Chinese medicine (TCM) has shown significant advantages in long-term clinical application and basic research. *Exocarpium Citri Grandis* (ECG; *Huajuhong* in Chinese) is the dried exocarp of Rutaceae plant Citrus Grandis ‘Tomentosa’ or Citrus grandis (L.) Osbeck (Committee for the Pharmacopoeia of People’s Republic of China, 2020). In TCM, ECG is widely used as a medicinal herb with the same origin as food and medicine to treat alcohol consumption and high fat [6, 7]. Research has shown that ECG has a good anti-inflammatory, lipid-lowering and antioxidant [8]. And ECG can alleviate the nonalcoholic fatty liver disease (NAFLD) by inhibiting lipid accumulation and inflammatory infiltration [9]. Through modern technology analysis, it has been confirmed that the active ingredients of ECG can reduce blood lipid levels and oxidative stress [2]. Although ECG has a potential role in lowering lipids and countering the effect of drinking, the exact therapeutic effects and underlying molecular mechanisms of ECG on ALD are still unclear.

ALD is recognized as a progressive condition, commencing with acute ethanol intake triggering oxidative stress and inflammation in the liver [10]. Prolonged overconsumption of ethanol then disrupts lipid metabolism, leading to excessive lipid accumulation [11, 12]. Clinical investigations have underscored that ALD is consistently accompanied by dysregulated lipid metabolism in the liver, characterized by lipid droplet accumulation in hepatocytes and modest inflammatory infiltration. This phenotype is prevalent in approximately 90% of heavy drinkers and may manifest within just two weeks following excessive alcohol intake [13]. Alarmingly, about one-third of patients with fatty liver, witness a worsening of liver inflammation, which is one of the unfavorable factors that aggravate the progression of ALD [14]. This persistent inflammation not only exacerbates the condition but also fosters the progression towards liver fibrosis and ultimately, cirrhosis [15]. Therefore, therapeutic strategies for ALD necessitate a dual focus: modulating lipid metabolism in hepatocytes to mitigate fat accumulation and controlling the excessive activation of immune cells to hinder inflammation. Moreover, the accumulation of lipids and subsequent oxidative stress induce lipid peroxidation within the liver and iron overload, which exacerbate liver damage [5, 16]. Addressing these interconnected mechanisms is pivotal in alleviating the detrimental consequences of ALD on liver health.

The present study aims to evaluate the protective effects of ECG against ALD and elucidate the underlying mechanisms to reveal the complex pathophysiological mechanisms involved in the evolution of ALD. The research results will provide the clinical treatment of ALD with important experimental basis and new ideas, thereby promoting advances in the treatment of the field.

## Materials and methods

### Reagents and antibodies

*Exocarpium Citri Grandis* (ECG) was purchased from chinese medicine pharmacy of Nanfang hospital (Identification NO.: BX80621, Lot NO.: HX22H01, Guangdong, China). The mixed standards of ECG were purchased from Chengdu Must Bio-technology Co., Ltd. Polyene phosphatidylcholine (PPC) (H20059010) was purchased from Beijing Sanofi Pharmaceutical Co., Ltd. Antibodies for GAPDH (#8884), Anti-mouse IgG HRP-linked Antibody (#7076) and Anti-rabbit IgG HRP-linked Antibody (#7074) were from Cell Signaling Technology; IBA-1 (ab178847), SREBP1 (ab28481), RAGE (ab3611), TfR (ab84036), Tf (ab82411), Hepcidin (ab30760), 4-HNE (ab48506) were from Abcam. Antibodies for GPX4 (67763-1), FTL (10727-1), HO-1 (10701-1) were from Proteintech. p-NF-κB (82335-1-RR) was from Proteintech. Antibody for FPN1 (NBP1-21502) was from Novus Biologicals. Goat anti-Rabbit IgG Alexa Fluor™ 488 (#A-11034) were from Invitrogen.

### Preparation of ECG extract and UHPLC-HRMS analysis

**Fig. 1:**
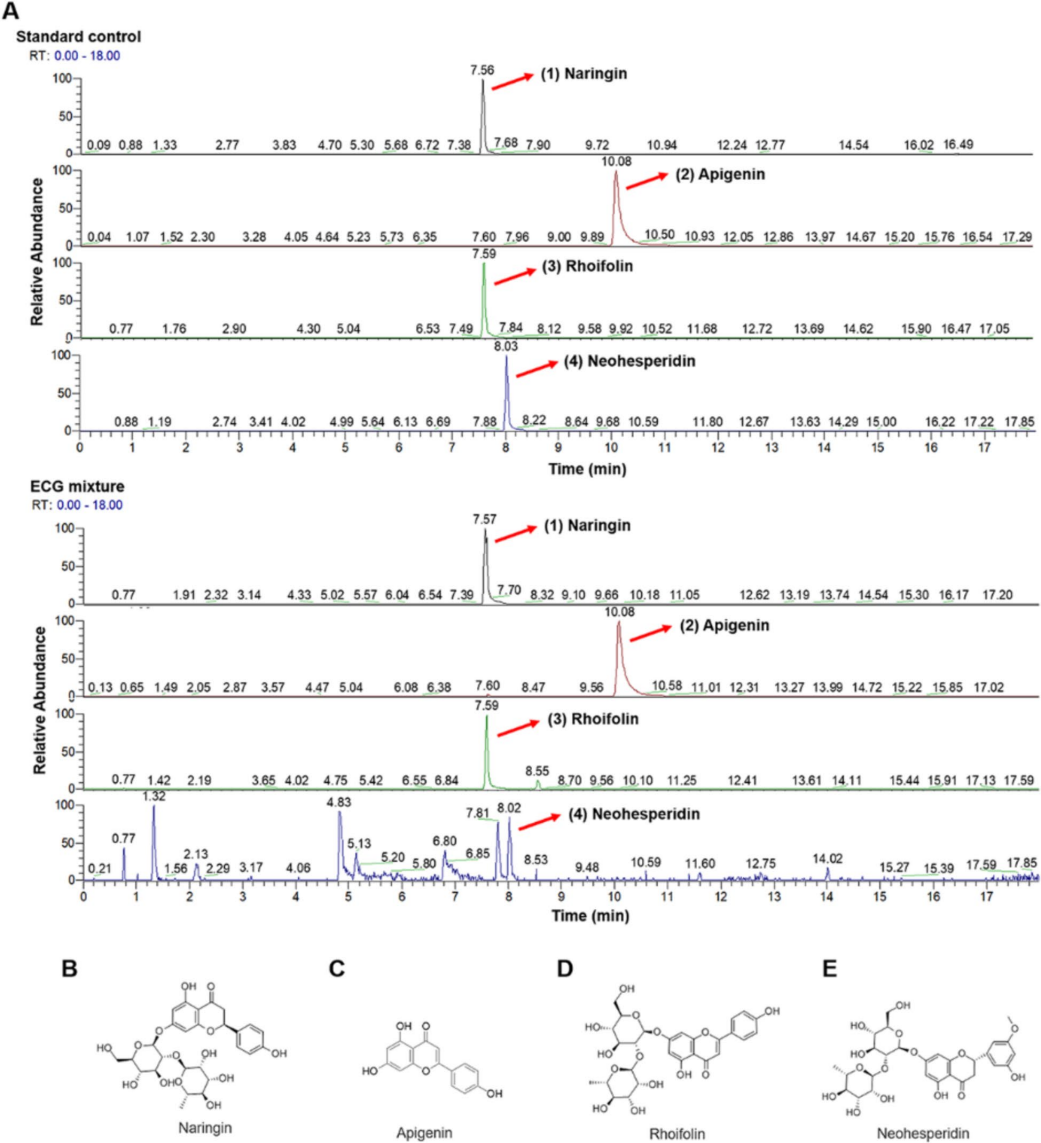
UHPLC-HRMS analysis of ECG extract. **A** UHPLC-HRMS tentative identification of active ingredients in ECG samples. UHPLC-HRMS chromatography of a standard mixture with 4 peaks determined by comparison of retention time with the ECG mixture: (1) Naringin; (2) Apigenin; (3) Rhoifolin; (4) Neohesperidin. **B–E** Chemical structures of the identified active ingredients

First, precisely weighed 200 g of ECG and added 12 times pure water to decoct the ECG for 1 h, repeated 2 times. Then, the ECG extract was concentrated to 1 g/mL and centrifuged at 3500 × rpm for 10 min to remove impurities. For quality control, 0.22 μm microporous membrane were used to pretreat ECG to filter impurities. The representative chemical components of ECG extract, Naringin, Apigenin, Rhoifolin and Neohesperidin (Fig. 1) were detected by ultra-high performance liquid chromatography-tandem high resolution mass spectrometry (UHPLC-HRMS) with a Hypersil gold 100*2.1, 1.9 μm column (ThermoFisher Scientific, Orbitrap Fusion Tribrid). The chromatographic conditions were as follows: 1. injection volume of 10 μl; 2. column temperature of 45 °C; 3. The mobile phase was eluted with 0.1% formic acid water (A) and acetonitrile (B) at a flow rate gradient of 0.3 mL/min. The elution process was as follows: 5% B at 0 min, 5% B at 1 min, 40% B at 10 min, 98% B at 16 min, and 98% B at 18 min.

### KEGG pathway enrichment analysis

The pathogenic genes of ALD were collected from Genecards database (https://www.genecards.org/). Protein coding genes were preserved. The chemical components of ECG were collected from HPLC. Similarity Ensemble Approach (SEA) was used to screen the targets of ECG components. The ECG targets and ALD pathogenic genes were used to contrasted PPI network based on STRING database with combine score > 00.4. The coregulation motifs is screened via the MCODE (2.0.3) plugin in Cytoscape (3.10.1). MCODE uses default analysis parameters (Degree cutoff = 2, Node Score cutoff = 0.2, K-Core = 2, Max. Depth = 100). Finally, cluster Profiler (4.10.1) in the Bioconductor package (https://bioconductor.org/) based on the R language (4.3.2) was used for KEGG pathway analysis. FDR-adjusted P values were set at 0.05, as the cut-off criterion.

### Zebrafish maintenance and treatment

The zebrafish came from the China Zebrafish Resource Center, National Aquatic Biological Resource Center (CZRC/NABRC). Within the contemplation of this investigation, we employed both wild-type zebrafish of the AB strain and liver-specific eGFP transgenic zebrafish Tg (lfabp10α: eGFP). Zebrafish larvae were raised and randomly divided into five groups and given different treatments at 96–98 h post fertilization (hpf) as our previous study [17, 18]: the control group (raised in water), model group (treated with 2% alcohol), ECG group (ECG dissolved in water): treated with 2% alcohol then ECG. The final concentrations of ECG were from 2.5 to 128 mg/mL.

### Mice treatment

Male C57BL/6 mice (8–10 weeks old, weight 20–22 g) were raised in the SPF Experimental Animal Management Center of Southern Medical University and had free access to sterilised water. The model group (alcohol group) were fed with the Lieber-DeCarli alcohol liquid diet (5% vol/vol) for 6 weeks, while the control group (pair-fed group) were fed with the control liquid diet for 6 weeks [19]. In parallel studies, after 3 weeks of liquid feeding, mice were intragastrically administered ECG extract in high (H, 6.68 g/kg/d), medium (M, 3.34 g/kg/d) or low (L, 1.67 g/kg/d) doses once a day for 3 consecutive weeks. Food intake and body weight of mice were recorded once a week. All animal procedures were approved by Southern Medical University Experimental Animal Ethics Committee (resolution NO.: L2020043, dated June 9, 2020).

### Preparation of medicated serum

20 male Sprague–Dawley rats aged three months (220 ± 20 g) were randomly divided into two groups: the control group and the ECG group. ECG was administrated to the dose (6 g/kg/d) twice a day for 3 consecutive days, and equivalent volume of sterilized water was orally administered to the rats of the control group. Rats were locally anesthetized with pentobarbital sodium injection to collect blood through the abdominal aorta 1 h after the last administration. The serum was centrifuged at 3000 rpm for 15 min and incubated with water bath at 56 ℃ for 30 min to inactivate the complement, then filtered through a 0.22 µm filter. The drug-containing serum and control serum were prepared as our previous report [20].

### Cell culture and proliferation assay

The murine hepatocyte line (AML-12) and the mononuclear macrophages line (RAW264.7) were procured and cultivated in accordance with our prior methodologies. These cells were categorized into five groups: a control group, a model group, and three clusters experiencing ECG concentrations (2.5%, 5%, and 10%). An in vitro ALD model was established by stimulating cells with 400 mM alcohol for a continuous period of 48 h. Concurrently, cells belonging to the ECG groups were cultivated in the presence of 400 mM alcohol and ECG-containing serum (at concentrations of 2.5%, 5%, and 10%) over a similar time span. Measurement of cell vitality was carried out deploying the Cell Counting Kit-8 (CCK-8, Dojindo, Japan).

### Cell transfection

Cells were transfected with RAGE over-expression (OE) lentivirus particles (Jikai Gene Technology Co., Ltd.) to construct RAGE-modified cell lines and screened with 2 μg/mL puromycin (Solarbio, China) according to the instructions.

### Biochemical detections

Mice serum aspartate aminotransferase (AST) and alanine aminotransferase (ALT) were detected by the biochemical kits (Nanjing Jiancheng Bioengineering insititue, Nanjing, China). Serum triglyceride (TG), total cholesterol (TC) and iron expressions were analyzed using automatic analysis biochemical analyzer (Shenzhen Lei Du Life Technology: Chemray 800 or Chemray 240). The expressions of superoxide dismutase (SOD), glutathione (GSH), glutathione peroxidase (GPx) and malondialdehyde (MDA) in the liver were measured by commercial reagent kits (Beyotime Biotechnology, Shanghai, China). Iron concentration in liver tissues were detected by iron assay kit (Abcam, ab83366) according to the instruction.

### Lipid peroxidation fluorescent probe detection

In vitro, lipid oxidative degree was detected by C11-BODIPY 581/591 (D3861, CAS: 217075-36-0, Thermo Fisher Scientific). Removed the cell culture medium and added C11-BODIPY 581/591 probe (5 μM) to incubate for 30 min in the dark, then photographed by the confocal microscope (Nikon, Japan).

### Haematoxylin and Eosin

Zebrafish larvae or mice livers were preserved in 4% paraformaldehyde (PFA) at 4 °C for 24 h. Following fixation, the samples underwent a gradual ethanol-mediated dehydration process, were embedded in paraffin, and subsequently sectioned into 4 μm slices. These sections were then subjected to dewaxing and rehydration, succeeded by hematoxylin and eosin (H&E) staining. After additional dehydration, the specimens were sealed with a neutral gum and subsequently assessed through conventional histological examination under a light microscope (Nikon, Japan).

### Detection of lipids

The specific methods of Oil red O and Nile red staining have been described in our previous research [21].

### Immunofluorescence staining

The liver tissues were fixed in 4% PFA and permeated with sucrose solution before being embedded in OCT gel. The livers were then cut into 14 μm thick sections. After 2 h of blocking, sections incubated with TfR (rabbit, 1:200), FPN1 (rabbit, 1:200), HO-1 (rabbit, 1:400), IBA-1 (rabbit, 1:1000), or SREBP1 (rabbit, 1:100) primary antibodies overnight respectively. Then sections were incubated with secondary antibody for 2 h at RT after being washed. Finally counterstained with DAPI and sealed with anti-fluorescence quencher (Solarbio Life Science, China).

### Immunochemistry

The immunochemistry method for paraffin sections of mouse liver was described earlier [21]. In brief, sections were deparaffinized, rehydrated, recovered the antigen, and inactivated in 3% H_2_O_2_ methanol for 10 min. After being incubated with blocking buffer solution for 2 h at RT, the samples were treated with primary rabbit antibody including 4-HNE (rabbit, 1:50), SREBP1 (rabbit, 1:200), GPX4 (rabbit, 1:1000), Hepcidin (rabbit, 1:100), RAGE (rabbit, 1:200), NF-κB (rabbit, 1:250), overnight at 4 °C. The slices were incubated with secondary antibodies at RT for 2 h on the second day. Then stained with DAB and counterstained with hematoxylin, finally dehydrated and sealed.

### Western blotting analysis

The western blot analysis was performed as previously reported [21]. The protein membranes were treated with the primary antibody including SREBP1, TfR, Tf, FTL, HO-1, RAGE, IBA-1, FPN1, GAPDH, overnight at 4 °C and then the secondary antibody for 2 h at RT.

### Quantitative real-time PCR

The specific gene primers (Table 1) were designed and synthesized by Sangon (Sangon Bioengineering, Shanghai). Total RNA was isolated from liver tissue by Trizol Reagent and quantified by gel electrophoresis and DS-11 + Spectrophotometer (DeNovix, USA). RNA was reverse transcribed into cDNA by EVO M-MLV Reverse Transcription Kit (AG11728, China). The real-time PCR (RT-PCR) was carried out on the LightCycler 96 RT-PCR system (Roche, Switzerland) following the program: denaturation for 5 min at 95 °C, followed by 40 cycles for 10 s at 95 °C and 30 s at 60 °C, melting for 10 s at 95 °C, 60 s at 65 °C and 1 s at 97 °C. The relative expression of genes were calculated by the 2^−△△CT^ with GAPDH as the reference gene.

**Table 1:** Gene primer sequences

Gene	Sequence (5′−3′)
*TNF-α* FP	GCCTCCCTCTCATCAGTTCTATG
*TNF-α* RP	ACCTGGGAGTAGACAAGGTACAA
*IL-6* FP	AGACTTCCATCCAGTTGCCTTCTTG
*IL-6* RP	TCTGTTGGGAGTGGTATCCTCTGTG
*IL-1β* FP	GTATCACTCATTGTGGCTGTGGAG
*IL-1β* RP	CCAGCAGGTTATCATCATCATCCC
*CCL2* FP	TGGGCCTGCTGTTCACAGTT
*CCL2* RP	TTCTTTGGGACACCTGCTGCT
*iNOS* FP	CACAGTCCTCTTTGCTACTGAGACAG
*iNOS* RP	CAGTAGTTGCTCCTCTTCCAAGGTG
*GAPDH* FP	GACCTCAACTACATGGTCTACA
*GAPDH* RP	ACTCCACGACATACTCAGCAC

FP: forward primer; RP: reverse primer

### Statistical analysis

Statistical comparisons between two groups were conducted using the unpaired t-test. The comparisons among multiple groups were performed using one-way analysis of variance (ANOVA), followed by Tukey’s post-hoc test for pairwise comparisons. The significance of survival analysis was determined by the Kaplan–Meier method and the log-rank test. The values of the measurement data were presented as mean ± standard deviation (SD), and a P value < 0.05 was considered statistically significant. All statistical analyses were performed using GraphPad Prism 8.0 software (GraphPad Software Inc., USA).

## Results

### UHPLC-HRMS was used to ensure the quality control of ECG

In modern research, flavonoids, polysaccharides, coumarins, volatile oil, and other natural products have been isolated from ECG [2], among them, naringin, apigenin, Rhoifolin, neohesperidin and other flavonoids are the main active ingredients [9, 22]. Similar to existing studies, also included naringin, neohesperidin, rhoifolin and apigenin were detected by the UHPLC-HRMS, four of which were flavonoids (Fig. 1A). The chemical structures of the flavonoid ingredients of ECG were shown in Fig. 1B–E.

### ECG inhibited alcohol induced lipid accumulation in zebrafish liver

**Fig. 2:**
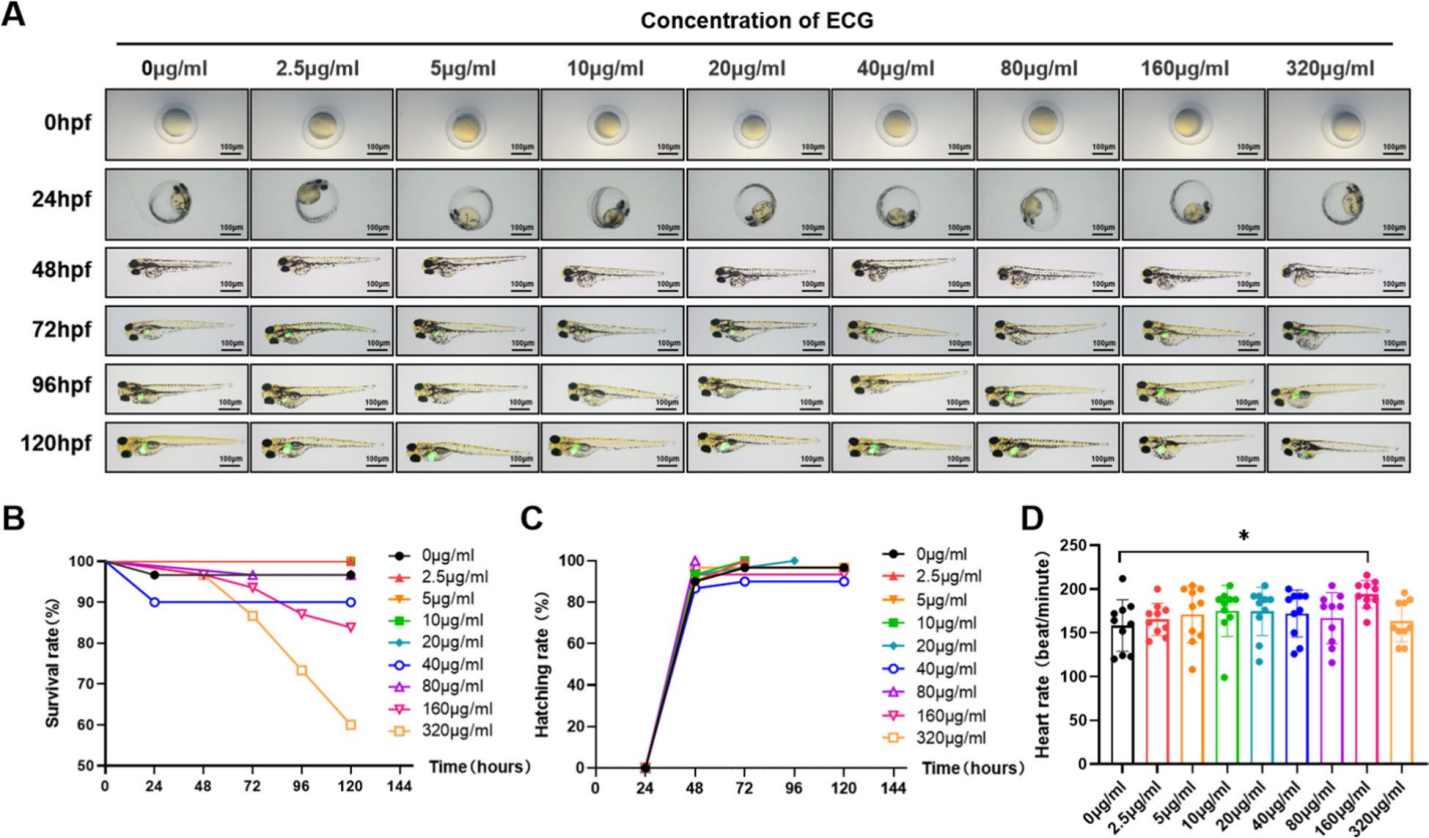
Toxicology of ECG in zebrafish larvae. **A** The morphology and liver development of zebrafish exposed to each concentration of ECG for 5 days (n = 6). **B** The survival rate of zebrafish at each concentration of ECG (n = 30). **C** Hatchability of zebrafish (n = 30). **D** Heart rate of zebrafish exposed to each concentration of ECG (n = 10). ^*^P < 0.05, ^**^P < 0.01, ^***^P < 0.001, ns = not significant

Zebrafish larvae at 8 hpf were exposed to ECG for a five-day consecutive treatment, and the toxicity of ECG on zebrafish larvae was observed through survival rate, morphological changes, heart rate and hatchability. There was no obvious morphological abnormality of zebrafish larvae in the concentration of 0–160 μg/mL ECG displayed by the images of zebrafish labeled with 1fabp10α-eGFP liver-specific fluorescence. But the production of hepatocytes and the absorption of yolk sac of zebrafish were decreased under 320 μg/mL ECG (Fig. 2A, S1A). Besides, the survival rates of zebrafish larvae treated with 0–80 μg/mL ECG for five days were more than 90%, while treated with up to 320 μg/mL ECG for four days, the survival rate of zebrafish was lower than 80% (Fig. 2B, S1B). The hatchability of zebrafish larvae could be maintained above 80% after incubating with 0–1 mg/mL ECG for 48 h (Fig. 2C, S1C). For heart rate, different concentrations of ECG have no notable influence on zebrafish larvae (Fig. 2D). Hence, based on the toxicity outcomes, ECG demonstrated low toxicity and was deemed relatively safe. Therefore, concentrations of 20, 40, and 80 μg/mL were selected for subsequent experimentation.

**Fig. 3:**
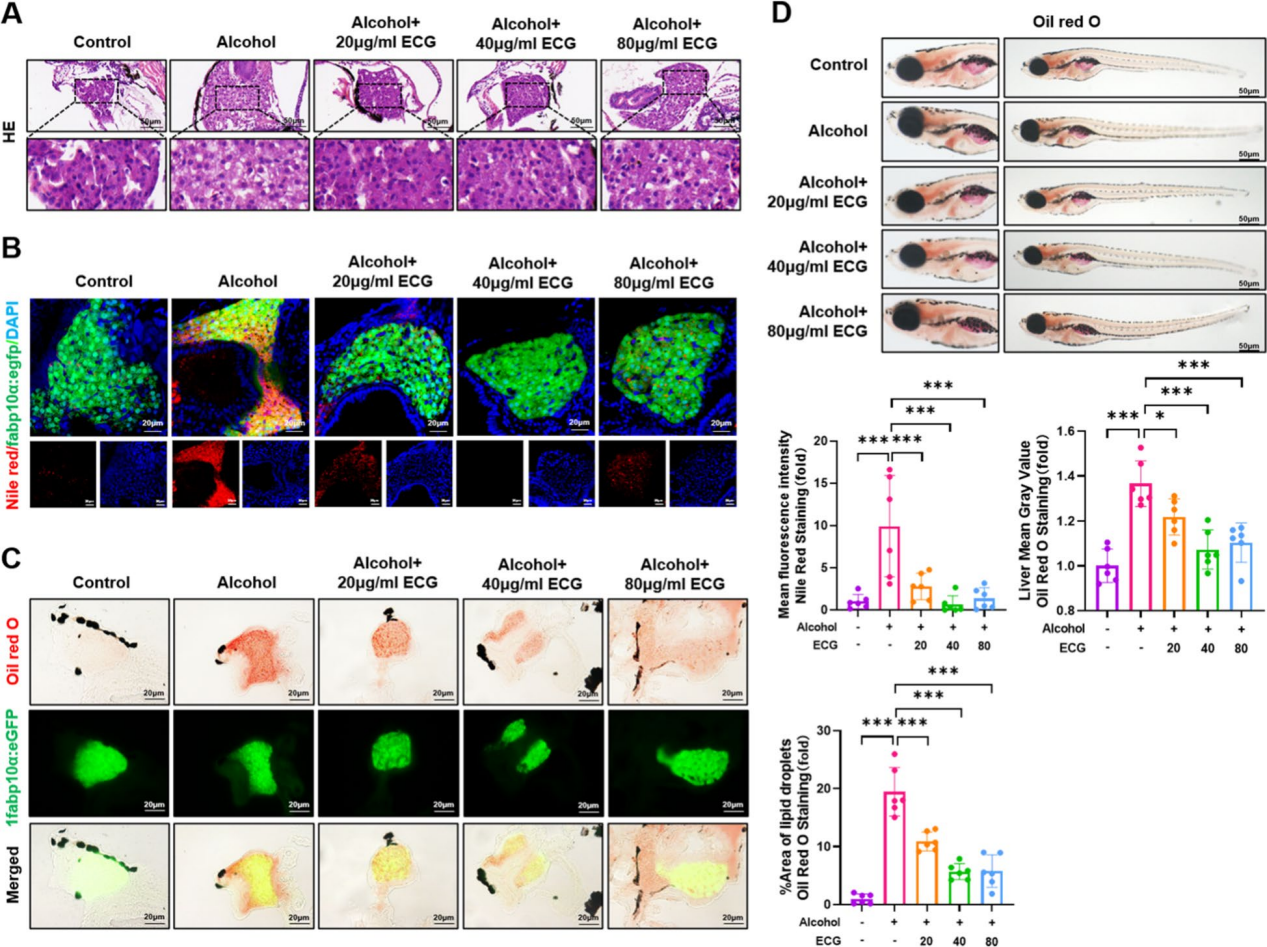
ECG reduced alcohol induced lipid accumulation in zebrafish liver. **A** H&E staining of zebrafish larvae (n = 6). **B, C** Nile Red and Oil red O staining of frozen sections of zebrafish with liver-specific green fluorescence expression. **D** Oil Red O staining of whole fish (n = 6). ^*^P < 0.05, ^**^P < 0.01, ^***^P < 0.001, ns = not significant

According to previous studies, an ALD model was effectively established by zebrafish larvae to 32-h stimulation with a 2% alcohol solution [17]. Hematoxylin and eosin (H&E) staining showed significant lipid accumulation and vacuolization in zebrafish livers in model group, while reduced in the ECG treatment group (Fig. 3A). The results of Nile red and Oil red O staining of zebrafish larvae showed that 20, 40 and 80 μg/mL ECG could reduce alcohol-induced lipid accumulation to different degrees (Fig. 3B,C). Similarly, whole-fish Oil red O also indicated a significant decrement in the degree of fatty liver within the ECG treatment groups in contrast to the model group (Fig. 3D).

### ECG ameliorated liver injury and abnormal lipid metabolism induced by alcohol diet in mice

**Fig. 4:**
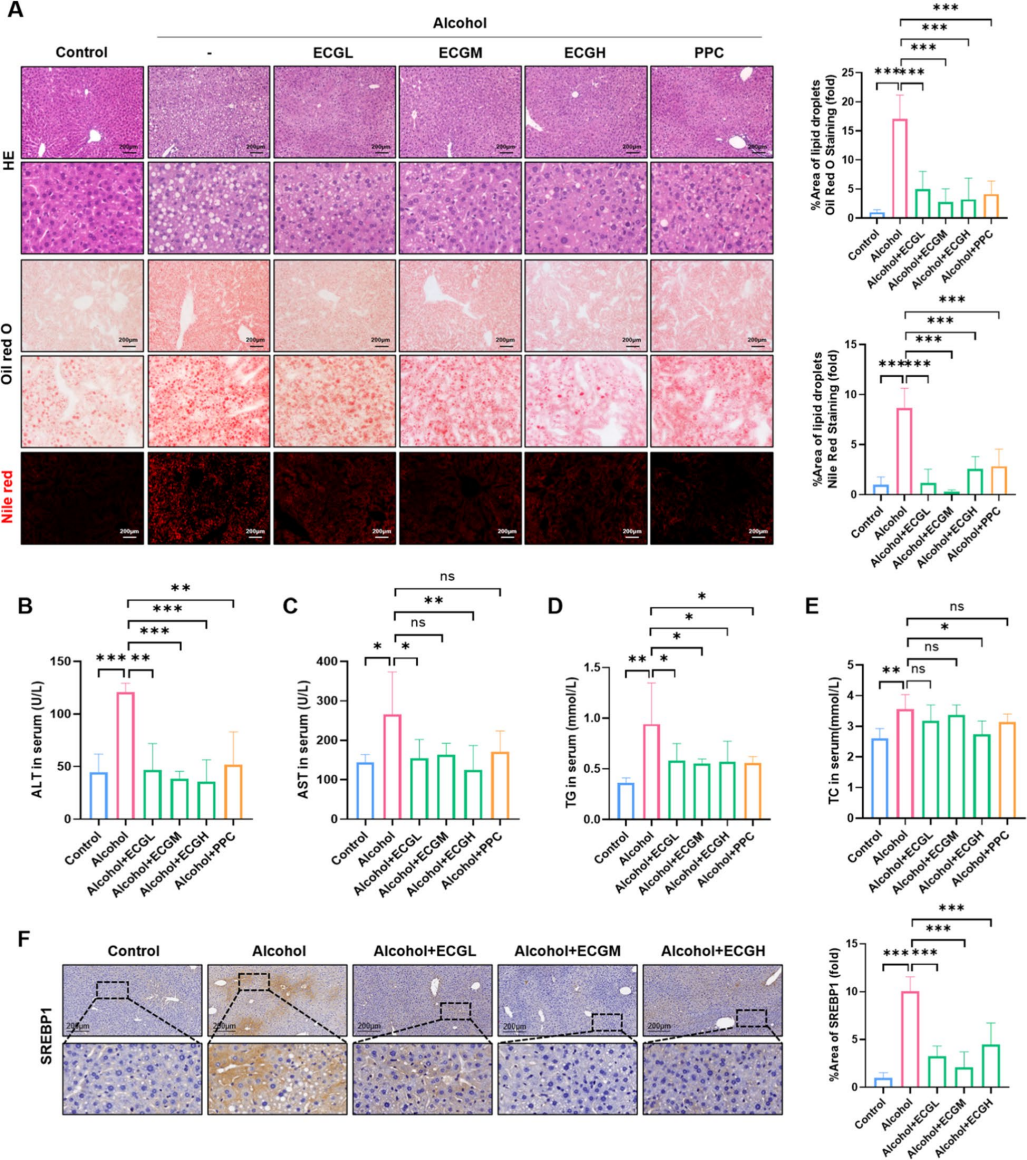
ECG alleviated lipid disorder and regulated lipid metabolism genes in alcoholic liver disease. **A** The lipid accumulation was visualized and quantified by mice hepatic H&E, Oil Red O and Nile Red staining (n = 4). **B, C** Serum ALT and AST expressions (n = 5). **D, E** Lipid expressions of TG and TC in mice serum (n = 5). **F** Immunochemistry staining for SREBP1 and quantitative analysis. ^*^P < 0.05, ^**^P < 0.01, ^***^P < 0.001, ns = not significant

To further explore the prevention and treatment effect of ECG on ALD, mice model was used for further study. H&E, Nile red and Oil red O stainings collectively demonstrated a significant reduction in the aggregation of vacuoles and lipid droplets in mouse liver tissues due to the effects of ECG extract (Fig. 4A). And ECG and PPC intervention decreased serum ALT and AST expressions (Fig. 4B,C). In addition, lipids in serum such as TG and TC were elevated in model group and decreased after ECG intervention (Fig. 4D,E), and the effect of high concentration group was better than the PPC group. Besides, immunohistochemistry showed that ECG reversed the alcohol-induced increase of SREBP1 expression in the mice model (Fig. 4F), indicating that ECG effectively reduced alcohol-induced liver lipid accumulation.

### ECG effectively suppressed liver inflammation triggered by RAGE activation in ALD

**Fig. 5:**
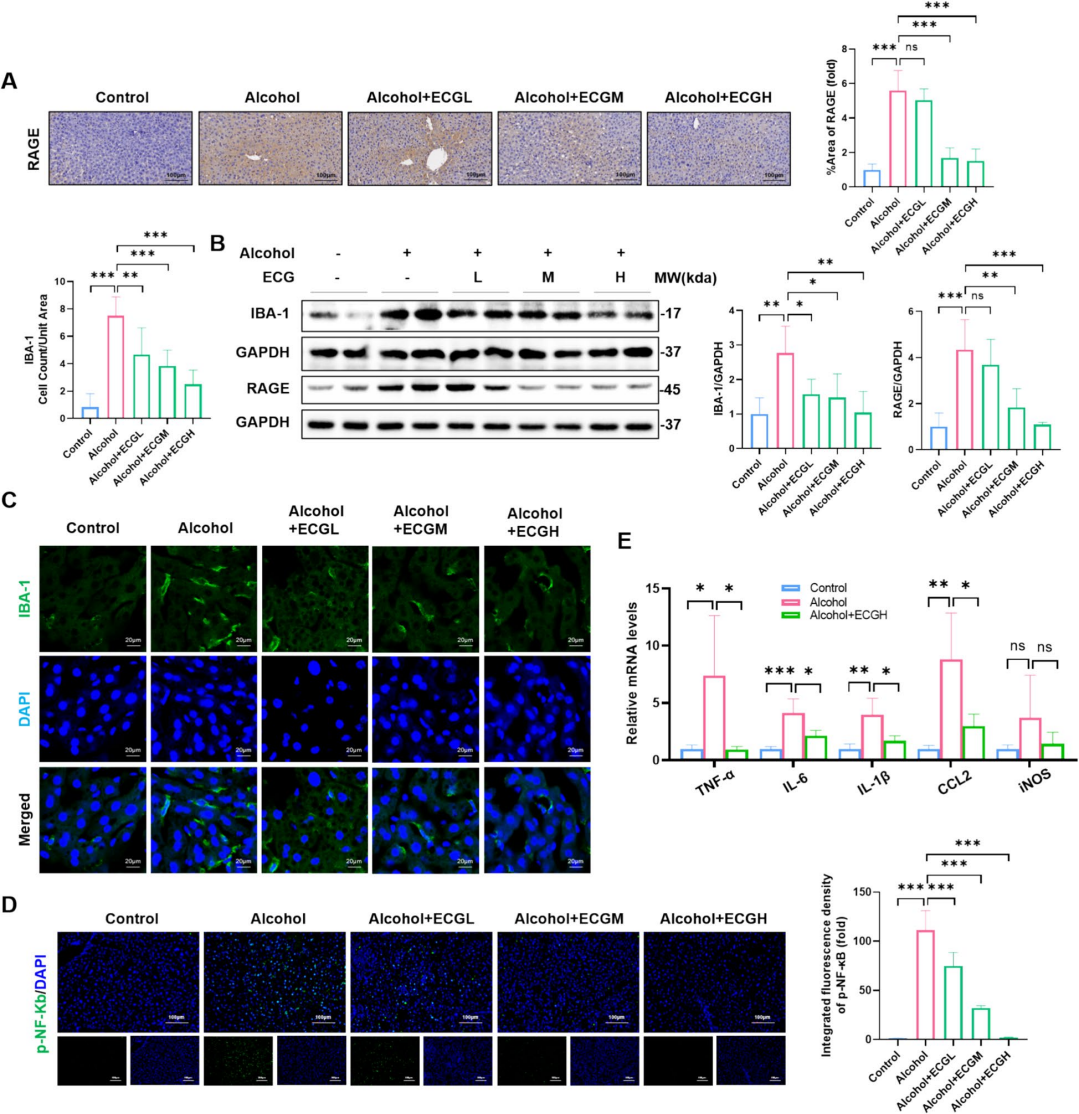
ECG suppressed liver inflammation triggered by RAGE activation in ALD. **A** Immunochemistry staining for RAGE and quantitative analysis (n = 6). **B** RAGE and IBA-1 expressions determined by western blot (n = 4). **C** Immunostaining for IBA-1 of mice liver tissues (n = 6). **D** Immunofluorescence for p-NF-κB in mice livers (n = 6). **E** The mRNA expression of liver TNF-α, IL-6, IL-1β, CCL2, iNOS genes were measured (n = 4). ^*^P < 0.05, ^**^P < 0.01, ^***^P < 0.001, ns = not significant

**Fig. 6:**
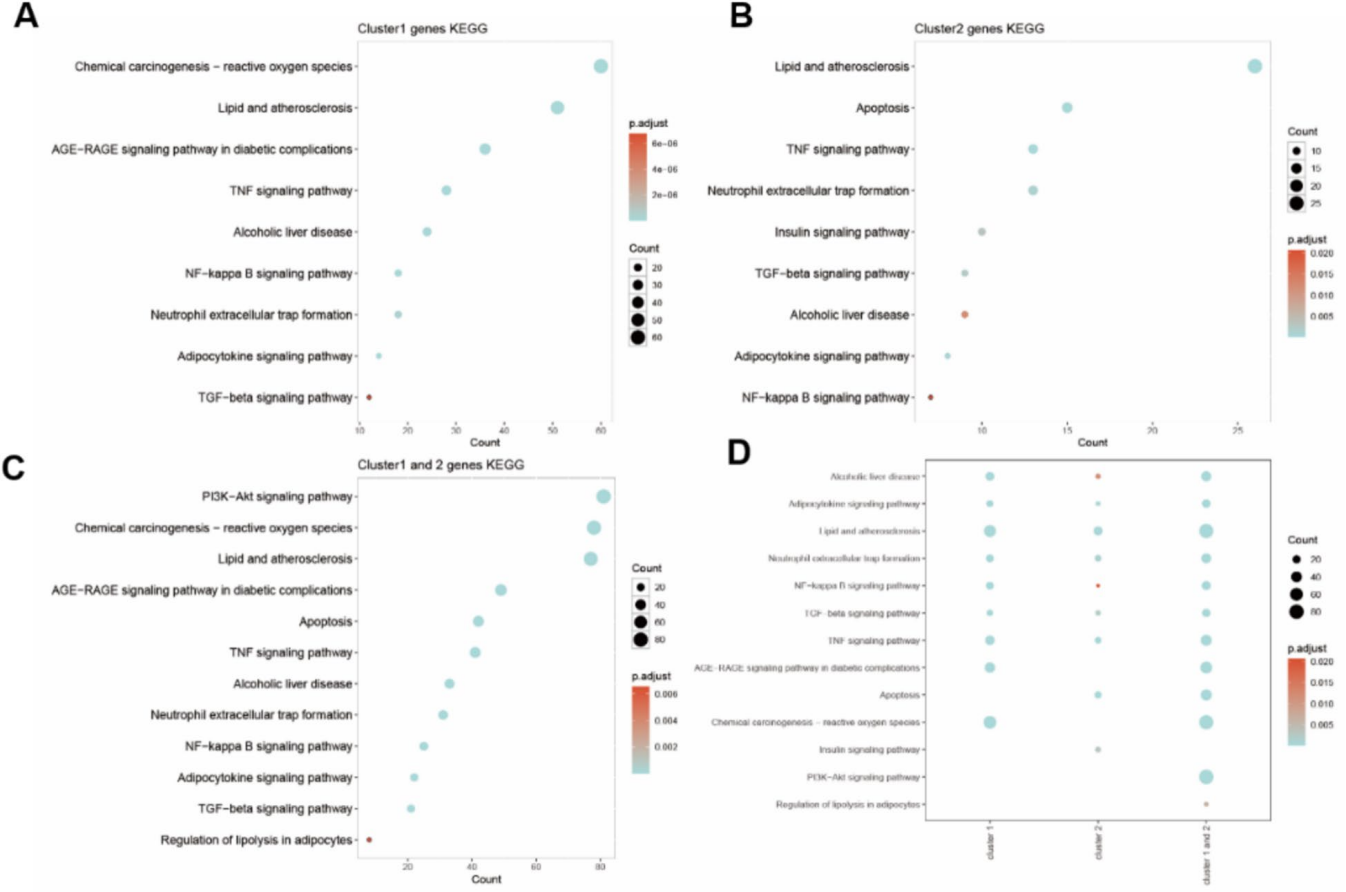
ECG target -ALD pathogenic gene: cluster1 gene -cluster2 gene -cluster1 and 2 gene KEGG bubble map and multi-group enrichment bubble map. **A** Cluster1 gene KEGG bubble map. **B** Cluster2 gene KEGG bubble map. **C** Cluster1 and cluster2 gene KEGG bubble map. **D** Cluster1 gene -cluster2 gene multi-group enrichment bubble map

Numerous clinical and experimental data have substantiated the prominent involvement of inflammatory cells, especially macrophages in the development and progression of ALD [23]. Immunofluorescence and western blot detections showed that ECG significantly inhibited the activation of alcohol-induced macrophages (marked by IBA-1) (Fig. 5B,C). We analyzed the interaction relationship between potential targets of ECG and pathogenic genes of ALD through the MCODE program and obtained the hub gene module network of the regulatory network3 (Figure S2). The enrichment analysis results of the KEGG pathways of the two regulatory networks show that the gene regulatory module not only plays a role mainly in the signaling pathways of “lipid metabolism” and “inflammation” such as TNFα, NF-κB in ALD, but is also highly correlated with the RAGE signaling pathway (Fig. 6). Studies have shown that RAGE can promote inflammation, cytokine synthesis and oxidative stress by activating NF-κB signaling pathway [24]. Immunohistochemistry and Western blot detects showed that ECG reversed chronic alcohol-induced upregulation of RAGE in the liver (Fig. 5A,B). The model group exhibited an upregulation of p-NF-κB expression and mRNA expressions of inflammatory factors such as TNF-α, IL-6, IL-1β, CCL2 and iNOS. However, these expressions were found to be decreased after treatment with ECG (Fig. 5D,E). The above results illustrated that ECG could inhibit macrophage activation and infiltration, thereby reducing alcohol-induced hepatic inflammatory injury, which might be related to the regulation of RAGE.

### ECG alleviated ALD by suppressing iron-dependent lipid peroxidation

**Fig. 7:**
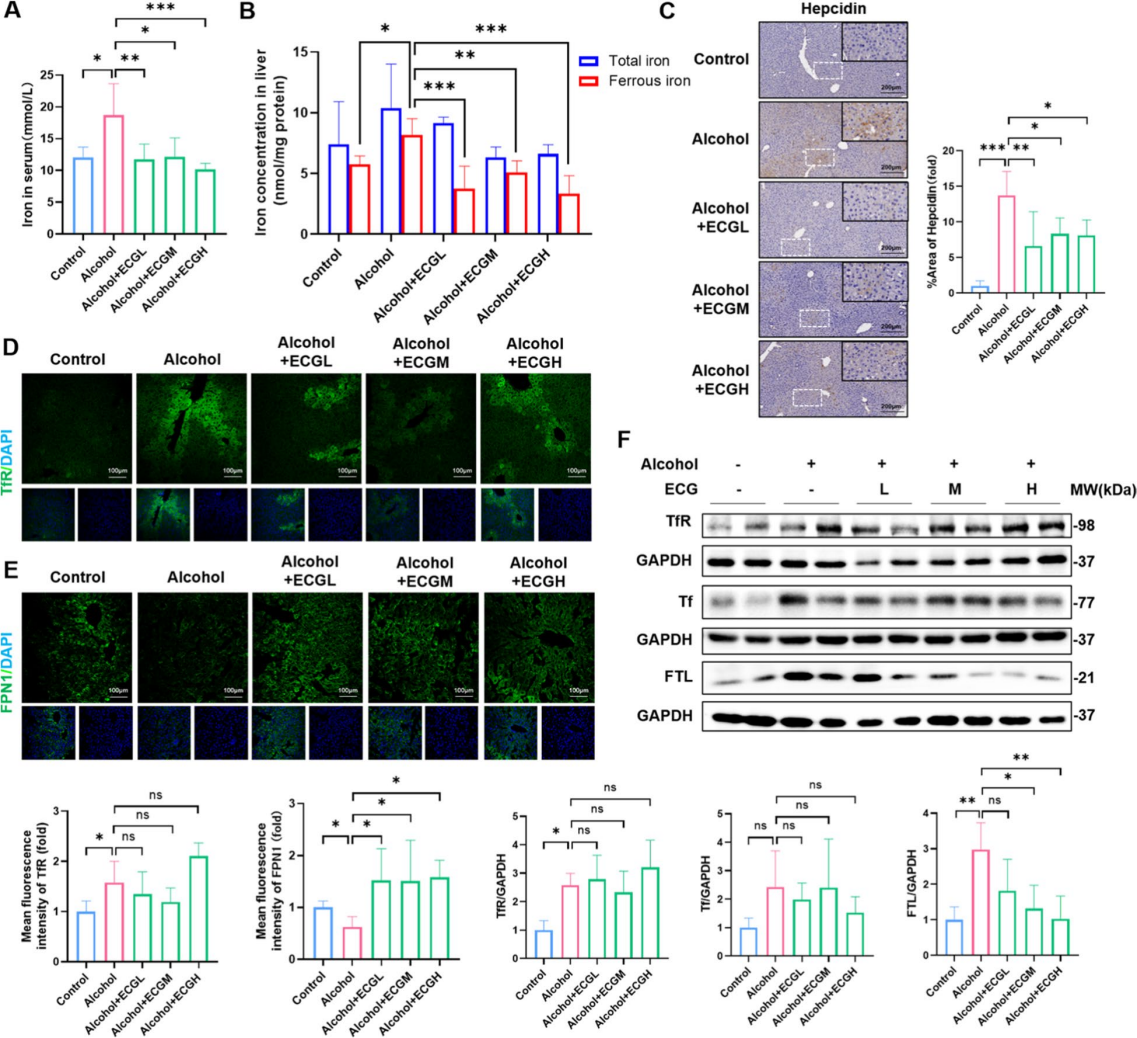
ECG inhibited alcohol-induced hepatic iron overload by regulating iron transport and storage pathways. **A** Serum expression of iron was measured (n = 5). **B** The concentration of total iron and ferrous iron in mice liver were detected (n = 5). **C** Immunohistochemistry of Hepcidin in mice livers (n = 6). **D, E** Immunofluorescence staining and quantitative analysis of TfR and FPN1 (green) (n = 5). **F** Hepatic TfR, Tf, and FTL expression tested by western blot (n = 4). ^*^P < 0.05, ^**^P < 0.01, ^***^P < 0.001, ns = not significant

Recent research has confirmed that RAGE plays a critical role in the regulation of iron metabolism in ALD. Genetic mutations or pharmacological inhibition of RAGE alleviated ALD by inhibiting inflammation and iron-dependent lipid peroxidation [21]. For investigating the regulatory effects of ECG on iron metabolism through RAGE signals in ALD, the content of iron and the expression of iron-related proteins were tested. Biochemical analyses revealed that in comparison to the control group, the expressions of both total iron and ferrous iron in serum and liver tissues were markedly elevated in the model group. However, these levers experienced a notable decline following the treatment of ECG (Fig. 7A,B), revealing that ECG reduced alcohol-induced cellular iron accumulation. Western blot, immunohistochemistry, and immunofluorescence staining showed a noteworthy upregulation of proteins involved in the Tf/TfR pathway, FTL and Hepcidin proteins, coupled with downregulation of FPN1 in the liver tissue of the model group. ECG partially inhibited the ferritin and Hepcidin proteins expressions and promoted the expression of FPN1 (Fig. 7C–F), although it had no significant effect on Tf/TfR pathway. These results demonstrated that ECG effectively inhibited iron-dependent lipid peroxidation, thereby alleviating alcohol-induced liver injury.

**Fig. 8:**
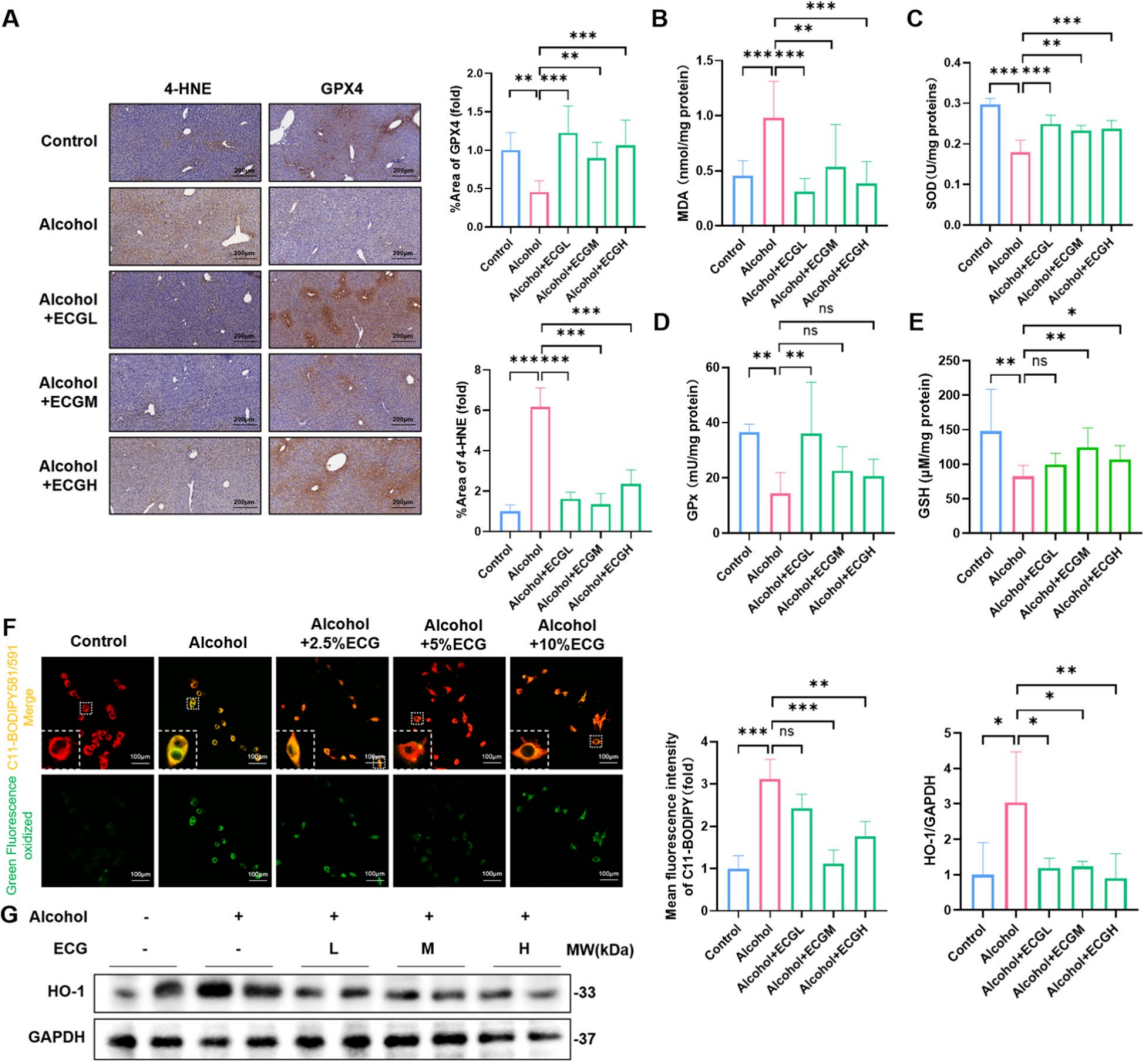
ECG inhibited lipid peroxidation in alcoholic liver disease. **A** Immunohistochemistry for 4-HNE and GPX4 of mice livers (n = 6). **B–E** The expressions of MDA, SOD, GPx and GSH were detected in mice livers tissues (n = 6). **F** Real-time observation of lipid peroxidation expression in AML-12 using C11-BODIPY 581/591 probe (n = 3). **G** HO-1 expression determined by western blot and the quantitative analysis (n = 4). ^*^P < 0.05, ^**^P < 0.01, ^***^P < 0.001, ns = not significant

Excessive iron usually results in lipid peroxidation, a well-established consequence of oxidative stress and is believed to be a crucial contributor to the pathogenesis of many diseases, including ALD. Immunohistochemical staining and biochemical tests showed that ECG inhibited the accumulation of 4-HNE and MDA in ALD (Fig. 8A,B). Meanwhile, the contents of GSH and SOD, and the activity of GPx in mice livers of the model group were decreased, while those of the ECG groups with different concentrations were increased (Fig. 8C–E). In addition, alcohol-treated AML-12 cells underwent severe lipid peroxidation as detected by C11-Bodipy 581/591 probe in vitro, while ECG intervention was significantly attenuated (Fig. 8F). Interestingly, heme oxygenase-1 (HO-1) was up-regulated in the model group, which contrasted with the suppression of another antioxidant protein GPX4 (Fig. 8A,G). Studies have shown that HO-1 expressed in macrophages can decompose heme to recover circulating iron, causing iron overload in the liver. These results implied that macrophages might participate in the iron homeostasis process in ALD.

### ECG differentially regulates iron metabolism in hepatocytes and macrophages

**Fig. 9:**
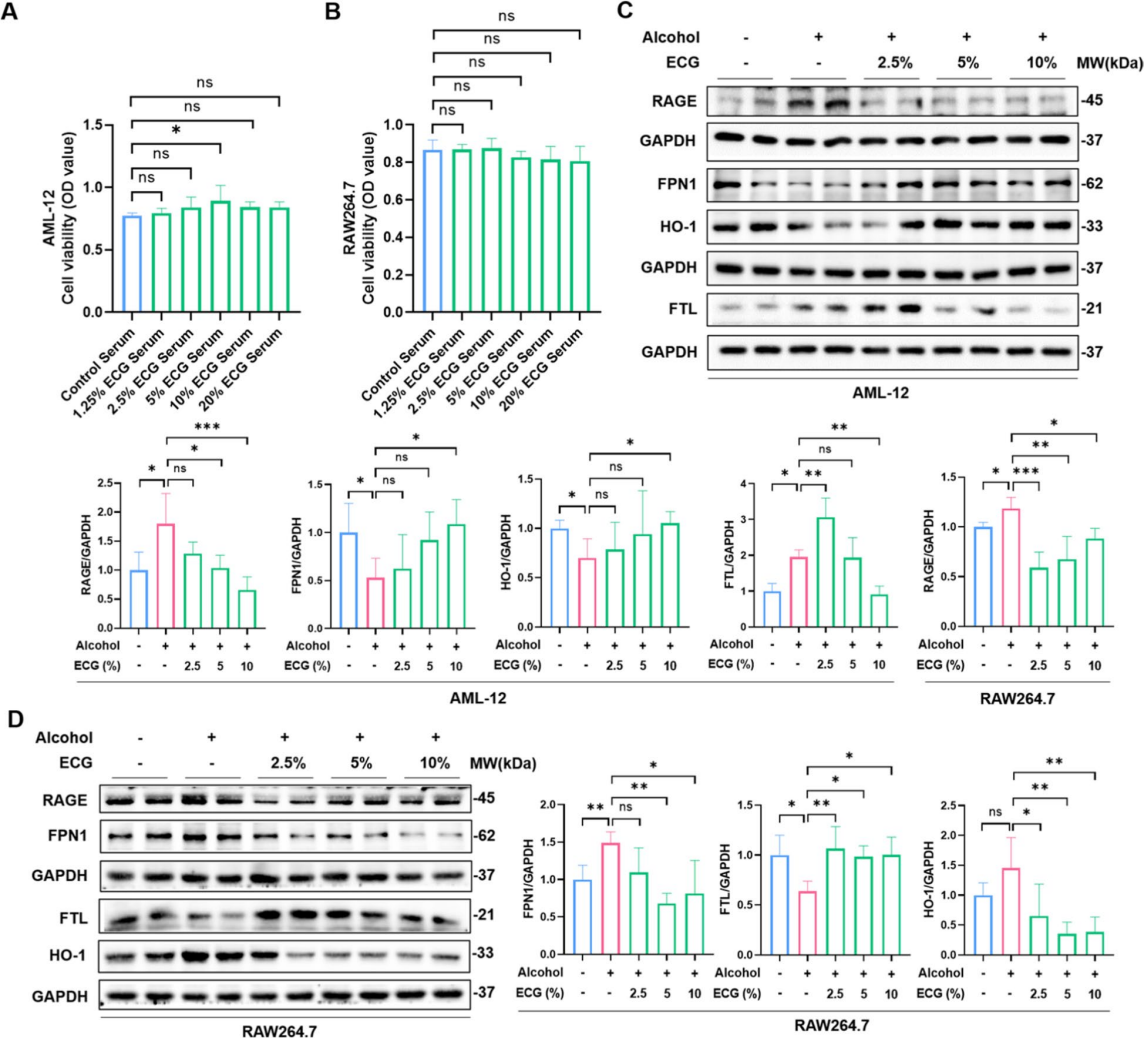
ECG exerted different regulatory effects on proteins related to iron metabolism on hepatocytes and macrophages. **A, B** Viability of hepatocytes and macrophages in different groups were tested by CCK8 assay (OD value was shown) (n = 6). **C** The expressions of RAGE, FPN1, FTL, HO-1 proteins were detected by western blot in AML-12 cells (n = 4). **D** The expressions of RAGE, FPN1, FTL and HO-1 proteins in RAW264.7 cells (n = 4). ^*^P < 0.05, ^**^P < 0.01, ^***^P < 0.001, ns = not significant

As we know, macrophages are the essential cell population for iron metabolism. The iron overload in hepatocytes of alcoholic steatohepatitis is partly due to the increase of iron released and decomposed by macrophages [25, 26]. To definitively determine the specific impact of ECG on iron metabolism within hepatocytes and macrophages during the progression of ALD, in vitro cells experiments were conducted. CCK8 showed ECG containing serum within 20% had no significant cytotoxicity on AML-12 and RAW264.7 cells (Fig. 9A,B). Both cells were stimulated with 400 mM alcohol for 48 h to construct ALD models as previously reported. Western blot result showed that ECG reversed alcohol-induced up-regulation of FTL and down-regulation of FPN1 and HO-1, indicating that ECG suppressed the increase in cellular iron storage and decrease in iron excretion induced by alcohol (Fig. 9C). For macrophages, FPN1 and HO-1 was up-regulated, and FTL was down-regulated after alcohol treatment, suggesting a decreased iron storage and an increased iron excretion in macrophages. And these results were reversed by ECG intervention (Fig. 9D). It is important to note that HO-1 was down-regulated in hepatocytes but up-regulated in macrophages after alcohol treatment. These results suggested that ECG could enhance the antioxidant capacity of HO-1 in hepatocytes and reduce the iron recycling capacity of HO-1 in macrophages. In addition, our recent study found that in ALD, RAGE has different regulatory effects on iron metabolism in hepatocytes and macrophages [21]. Herein, ECG effectively inhibited the expression of RAGE in both hepatocytes and macrophages when stimulated by alcohol, suggesting that ECG might affect the iron metabolism function of different cells by regulating RAGE signal.

### ECG mediated cellular iron metabolism was associated with RAGE expression

**Fig. 10:**
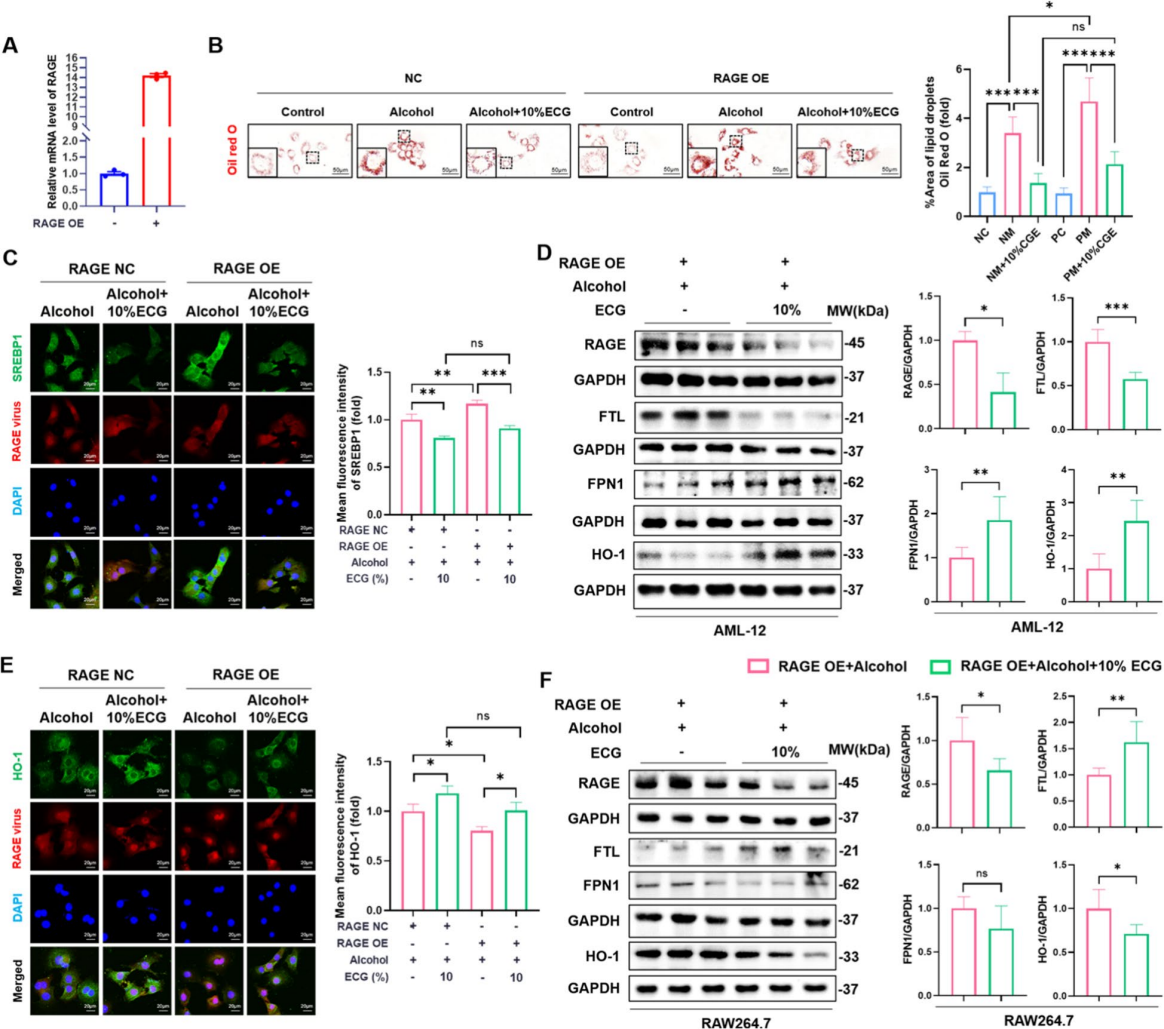
ECG alleviated liver injury by reversing the imbalance of cellular iron metabolism induced by RAGE overexpression. **A** RT-PCR analysis of the mRNA expression of RAGE in AML-12 cells (n = 3). **B** Evaluation of cells lipid droplets by Oil Red O staining (n = 4). **C** Immunofluorescence staining of SREBP1 (green). **D** The expressions of RAGE, FTL, FPN1 and HO-1 in AML-12 cells (n = 3). **E** Immunofluorescence staining of HO-1 (green). RAGE NC or OE virus were labeled with red fluorescent (n = 3). **F** Expressions of RAGE, FTL, FPN1 and HO-1 in RAW264.7 cells were detected by western blot (n = 3). ^*^P < 0.05, ^**^P < 0.01, ^***^P < 0.001, ns = not significant

To further study the relationship between ECG and RAGE, we first utilized lentivirus to construct the cell strain overexpressing RAGE (Fig. 10A). Cells were simultaneously added different concentrations of ECG for intervention. Western blot results showed that ECG intervention partially resisted RAGE overexpression in AML-12 and RAW264.7 cells (Fig. 10D,F). Oil Red O staining showed that lipid droplets increased in negative control (NC) + alcohol group compared with NC + control group, and were more evident in the RAGE OE + alcohol group. ECG intervention reduced alcohol-induced cellular steatosis in both NC and RAGE OE groups (Fig. 10B). Meanwhile, ECG inhibited the upregulation of SREBP1 induced by RAGE overexpression (Fig. 10C). For iron metabolism, western blot results showed that compared with RAGE OE + alcohol group, FTL decreased in RAGE OE + ECG group, while HO-1 protein was significantly increased, although FPN1 was only slightly upregulated (Fig. 10D,E), suggesting that a decrease of iron storage and an increase of iron export in hepatocytes. For macrophages, compared with RAGE OE + alcohol group, FTL increased in RAGE OE + ECG group, while HO-1 and FPN1 were decreased (Fig. 10F), prompting macrophages to store more iron and suppress iron recycling and export to avoid excessive iron intake by hepatocytes subsequently. ECG intervention effectively reversed the dysfunction of cellular lipid and iron metabolism mediated by RAGE overexpression.

To further investigate whether RAGE is a target for ECG, we used Autodock Vina to investigate the possible interaction between the active ingredients of ECG and RAGE. The results of the study exhibited that the binding energy of the naringin-RAGE complex, apigenin-RAGE complex, rhoifolin-RAGE complex and neohesperidin-RAGE complex, were − 7.6 kcal/mol, − 6.5 kcal/mol, − 6.9 kcal/mol and − 5.4 kcal/mol, respectively. The 3D docking conformations of the active ingredients-RAGE complex were shown in Figure S3. Molecular docking analysis showed that naringin could interact perfectly with four amino acid residues of RAGE (PRO-80, VAL-78, SER-65, ARG-77) through hydrogen bond, apigenin could interact with two amino acid residues of RAGE (ARG-57, THR-58) through hydrogen bond, rhoifolin could interact with five amino acid residues of RAGE (ARG-114, ARG-116, GLU-59, GLY-56, ARG-57) through hydrogen bond, and neohesperidin could interact with three amino acid residues of RAGE (THR-109, ASN-112, GLN-24) through hydrogen bond (Figure S3A-D). These results suggested that RAGE could effectively combine with ECG.

## Discussion

**Fig. 11:**
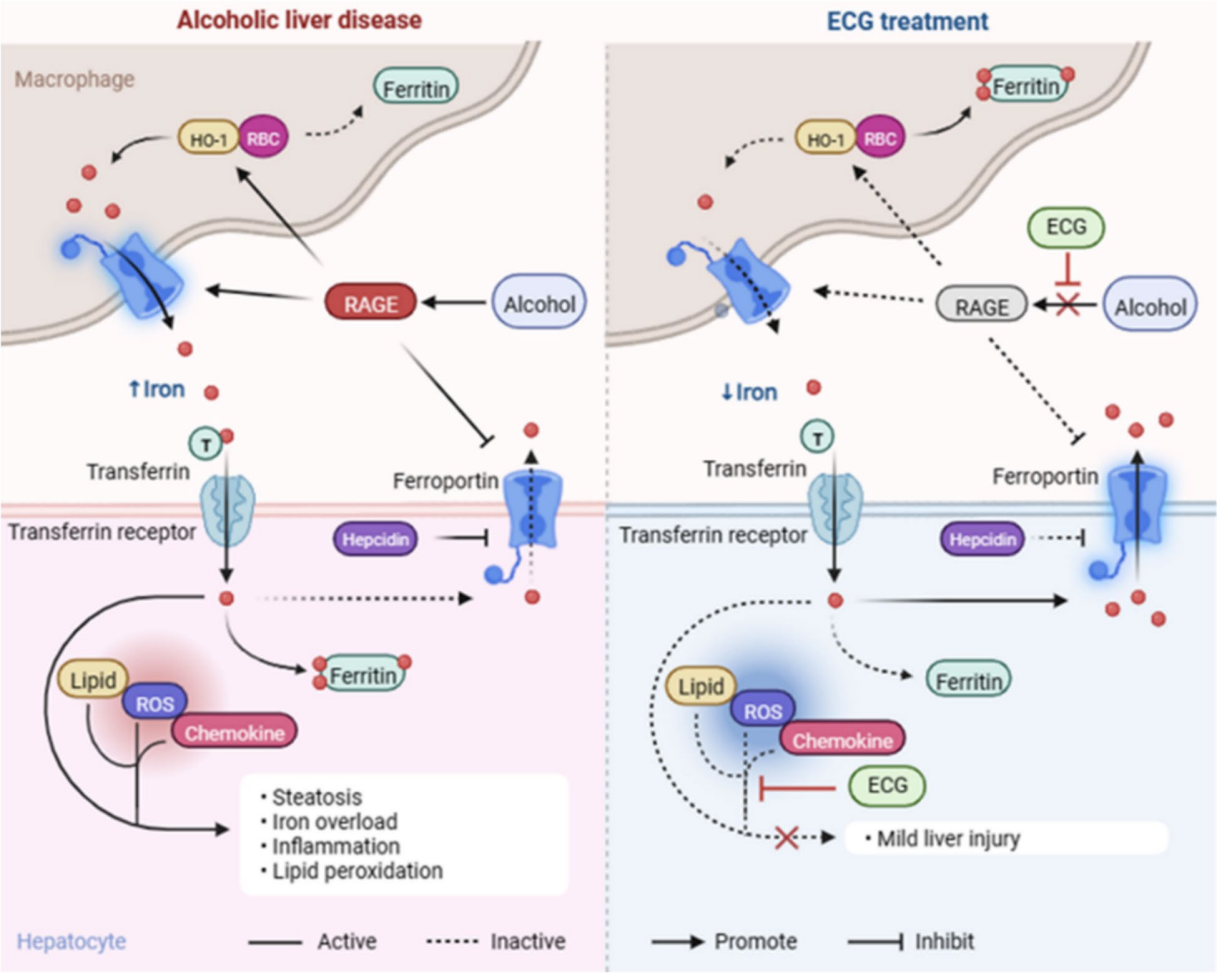
Schematic diagram showing the potential mechanism related to the therapeutic effect of ECG on ALD. (Created with BioRender)

The increased alcohol consumption has led to a significant rise in the global incidence rate and mortality [27]. Currently, the Food and Drug Administration (FDA) has not approved any efficacious drugs that have been shown to be effective in patients with ALD [27]. Therefore, it is essential to understand the role of hepatotoxic intermediates in the progression of ALD and to provide clues for formulating treatment strategies (Fig. 11).

In recent years, zebrafish has emerged as a novel animal model for drug discovery and medical research due to its unique biological characteristics [29]. Compared with the mouse models, the zebrafish offers distinct advantages, including rapid ontogenesis, exceptional suitability for high-throughput screening, and compatibility with in vivo high-resolution imaging of live embryos through integration of fluorescent labeling and confocal microscopy. There are a variety of zebrafish species labeled with cell fluorescence, which can quickly visualize their composition, function, signal transduction, damage response and other physiological and pathological processes of liver cells. Through high-throughput screening of fluorescently labeled zebrafish, we found that the drug toxicity of ECG was low in various aspects, such as morphology, heart rate, hatching rate and survival rate. Furthermore, ECG significantly reduced alcohol-induced lipid accumulation in zebrafish liver. Chronic alcohol intake has been shown to induce hepatic expression of SREBP1, a transcription factor responsible for promoting the expression of lipogenic genes, consequently leading to an elevation in fatty acid synthesis [30]. Simultaneously, we successfully constructed the ALD model in mice by feeding them the classic Lieber-Decarli diet for six weeks. ECG intervention effectively inhibited liver steatosis and elevated blood lipids in mice by regulating lipid metabolism gene SREBP1.

Macrophage is a significant component of immune response molecules released by the immune system. RAGE is an immunoglobulin-like receptor, which is mainly expressed in hepatocytes and macrophages. Alcohol intake can increase the expression of RAGE ligands in the liver and adipose tissue [31]. The results of KEGG pathway enrichment analysis showed that ECG exhibits characteristics of potential drug for the treatment of ALD. Its therapeutic mechanism is highly correlated with the RAGE signaling pathway and involves multiple processes such as regulating lipid metabolism and exerting anti-inflammatory effects, thereby effectively alleviating the progression of ALD. RAGE could induce the activation of the NF-κB signaling pathway and lead to severe liver inflammation, which further aggravates the fatty liver process. NF-κB is a redox-sensitive transcription factor, while p-NF-κB is its active form after activation. Once activated in the cell nucleus, it can stimulate the transcription of pro-inflammatory cytokines including TNF-α and IL-6. The outcomes of our research have conclusively demonstrated that ECG intervention suppresses the activation of hepatic macrophages by inhibiting the RAGE/p-NF-κB signaling, and further reduces the expression of cytokines such as TNF-α, CCL2, IL-6 and IL-1β, thereby alleviating alcohol-induced liver injury.

Clinical studies have shown that abnormal iron distribution in the liver is closely related to the incidence of ALD. Excessive chronic alcohol consumption is associated with elevated serum ferritin concentrations and transferrin saturation, leading to increased hepatic iron deposition. New reports suggest that iron deposition may predict death from hepatocellular carcinoma in patients with alcoholic cirrhosis [32]. And iron overload has been shown to be associated with macrophage activation and subsequent inflammation. Excess iron in mice increases the mRNA expression of pro-inflammatory cytokines such as IL-1β and IL-6 [33]. Besides, our recent research has substantiated that RAGE promoted ALD progression by stimulating inflammation and iron dependent lipid peroxidation. RAGE promoted ALD progression by stimulating inflammation and iron-dependent lipid peroxidation [21]. In this study, we found that ECG effectively inhibited the up-regulation of RAGE in ALD, suggesting that ECG might suppress liver iron-deposition by inhibiting RAGE and subsequently alleviating inflammatory infiltration. We further explored the mechanism of iron overload in chronic ALD. Our results suggested alcohol induced elevation of serum iron and liver iron in mice, which was reversed by ECG treatment. In general, the main route of iron entry into hepatocytes is the classical Tf/TfR mediated endocytosis system in all cells [34]. Hepatocytes store iron in ferritin and release it through the action of FPN1 [35]. However, the export of cellular iron can be inhibited by hepcidin, which binds to and triggers the degradation of FPN1 [36]. To further investigate the effect of ECG on iron overload in ALD, we found that ECG inhibited the upregulation of FTL and Hepcidin, and suppressed the downregulation of FPN1 induced by alcohol, although it did not significantly affect the Tf/TfR pathway. These findings illustrated that ECG might alleviate the progression of ALD by affecting the liver’s ability to store and transport iron.

Studies have shown that fatty acids containing bis-allylic carbons are highly sensitive to lipid peroxidation [37]. Lipid peroxidation and deleterious ROS, resulting from the iron-mediated Fenton reaction and enzymatic oxidation compound the damage induced by alcohol on liver tissues. The antioxidant function mediated by the GSH/GPX4 axis is essential in antagonizing the production of specific phospholipid hydroperoxides catalyzed by active iron [36]. Our results suggested that alcohol caused severe lipid peroxidation in the liver, which was manifested by the inhibition of GSH/GPX4 axis, the decrease of SOD and GPx activities, and the accumulation of lipid peroxidation products MDA and 4-HNE. Moreover, the intervention of different concentrations of ECG effectively reversed these pathological changes, revealing that ECG could resist iron-dependent lipid peroxidation in ALD. Interestingly, the antioxidant protein HO-1 was down-regulated after ECG intervention. HO-1, a member of the heat shock protein family, has been implicated in cellular antioxidant defense and anti-apoptotic functions [38]. In addition, HO-1 helps macrophages acquire iron by degrading heme and thus participates in iron metabolism. In this study, we proposed the hypothesis that the observed alterations in HO-1 expression within hepatic cells hinge upon its manifold functionality in various cellular contexts.

In combination with the previously discovered alteration in HO-1 and the possibility of macrophages participating in iron metabolism, we further verified our speculation through in vitro experiments. Encouragingly, we found that the ECG-mediated roles of hepatocytes and macrophages in iron metabolism were not identical. For macrophages, ECG promoted iron storage and inhibited iron export to reduce subsequent hepatocyte intake. For hepatocytes, ECG could promote iron excretion and inhibit iron storage. Therefore, we considered whether ECG could affect the function of iron metabolism in hepatocytes and macrophages by regulating RAGE respectively. As expected, ECG reversed macrophage activation and iron overload induced by RAGE overexpression (reduced acquisition and storage, and increased export). Simultaneously, ECG reversed the lipid accumulation and iron deposition induced by RAGE overexpression in hepatocytes (increased storage and decreased export). Additionally, molecular docking results also clarified the interaction between RAGE and the active ingredients of ECG. In summary, our findings demonstrated that ECG exerted protection against alcohol-induced ALD in vivo and in vitro. The protective effects of ECG might be related to the suppression of RAGE-mediated lipid accumulation and iron overload, as well as oxidative stress and the inflammation (Fig. 10). These data may offer novel insights into the pharmaceutical value of ECG in preventing and treating ALD. And deeper mechanisms need to be further studied in the future.

## Conclusions

Taken together, the current study provided the evidence that the natural medicine ECG had a therapeutic action in preventing liver injury induced by alcohol in vivo and in vitro. The protective effect might be related to the inhibition of RAGE-mediated lipid and iron accumulation, and subsequently suppressing the lipid peroxidation and inflammation. These findings provide the therapeutic promise of ECG in alcohol-induced liver injury.

## Supplementary Information

Additional file 1

## Data Availability

No datasets were generated or analysed during the current study.

## Abbreviations

ECG: *Exocarpium Citri Grandis*
TCM: Traditional Chinese Medicine
AST: Aspartate transaminase
ALT: Alanine aminotransferase
RAGE: Receptor for advanced glycation endproducts
ALD: Alcoholic liver disease
FPN1: Ferroportin 1
HO-1: Heme oxygenase-1
TC: Total cholesterol
TG: Triglyceride
FTL: Ferritin light chain
Tf: Transferrin
TfR: Transferrin receptor
4-HNE: 4-Hydroxynonenal
MDA: Malondialdehyde
SOD: Superoxide dismutase
GSH: Glutathione
GPx: Glutathione peroxidase
CCL2: Chemokine ligand 2
H&E: Hematoxylin and Eosin
SREBP1: Sterol-regulatory element-binding protein 1
CCK8: Cell counting kit 8
UHPLC-HRMS: Ultra-high performance liquid chromatography-tandem high resolution mass spectrometry
hpf: Hours post fertilization
TNF-α: Tumor necrosis factor-α
IL-6: Interleukin-6
p-NF-κB: Phosphorylated-nuclear factor-κ-gene binding
PPC: Polyene phosphatidylcholine
