# Supplementary material for: Exocarpium Citri Grandis ameliorates alcoholic liver disease by modulation of hepatic lipid metabolism and iron homeostasis
Source: Chin Med. 2025 Oct 16;20:174. doi: 10.1186/s13020-025-01229-4 (PMC12529825; doi:10.1186/s13020-025-01229-4)
Supplement: Supplementary file 1 — Additional file 1 [file 13020_2025_1229_MOESM1_ESM.docx]

**Supplementary materials**

**
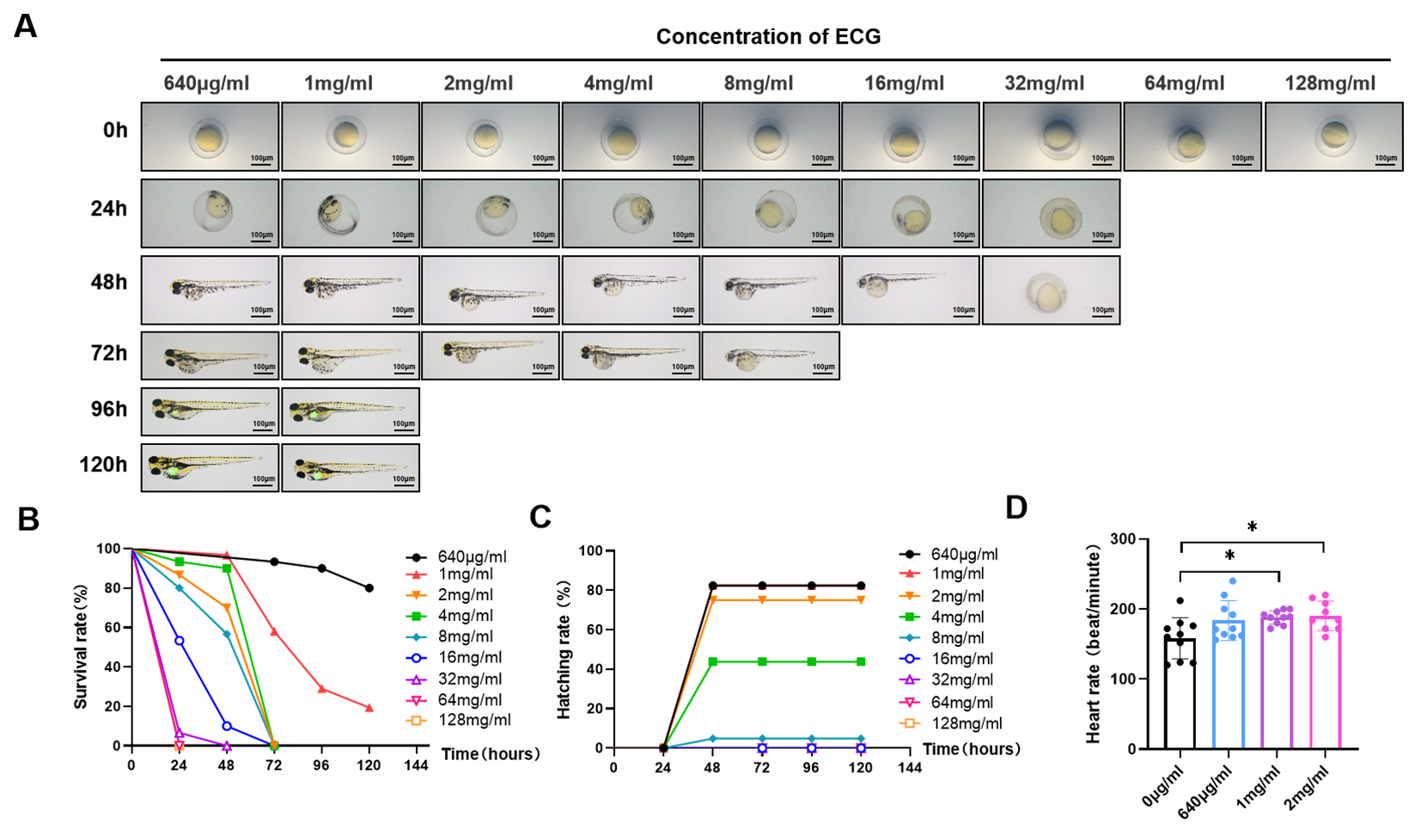
**

**FigureS1. Toxicology of ECG in zebrafish larvae.** (A) The effect of zebrafish larvae exposed to different concentrations of ECG for 5 days on zebrafish larval morphology and liver development (n=6) (B) The effect of different concentrations of ECG on the survival rate of zebrafish larvae (n=30). (C) Hatchability of zebrafish larvae (n=30). (D) Heart rate of zebrafish larvae exposed to different concentrations of ECG (n=10). The data are shown as mean ± SD (^*^*P*<0.05, ^**^*P*<0.01, ^***^*P*<0.001, ns=not significant).


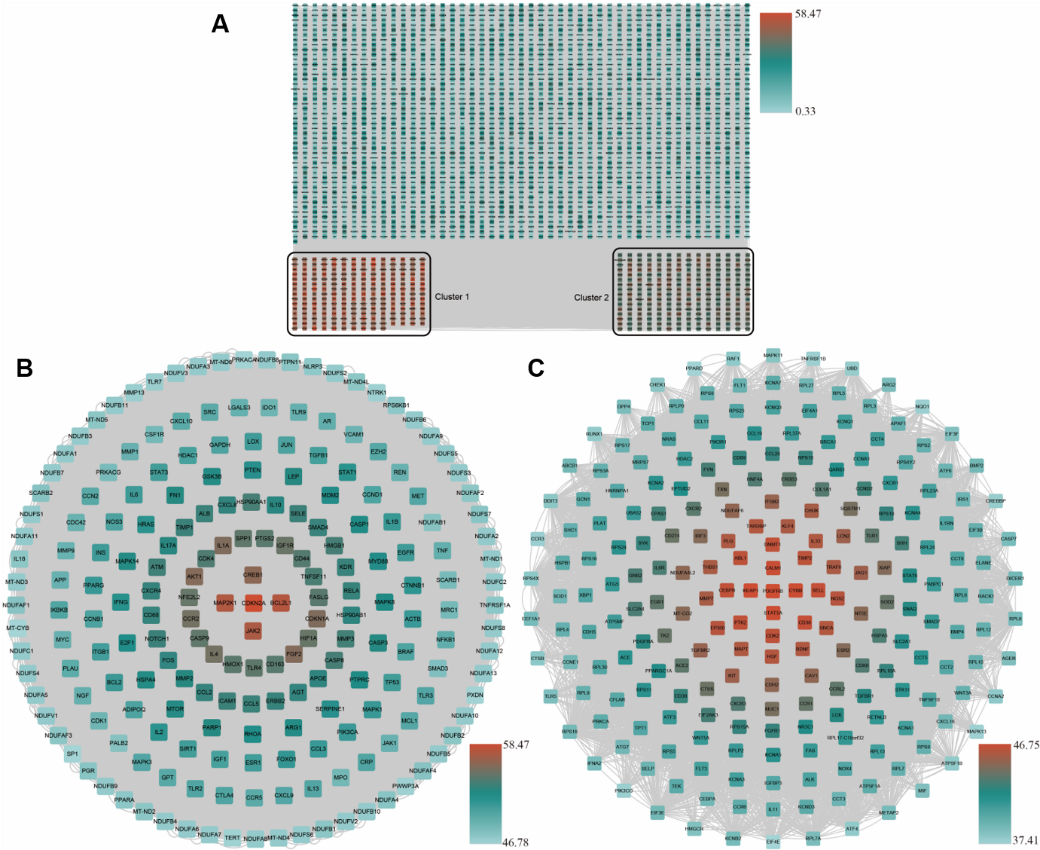


**FiguresS2. ECG target -ALD pathogenic gene -PPI network -MCODE analysis.** (A) ECG target-ALD pathogenic gene-MCODE analysis. (B) ECG target -ALD pathogenic gene - PPI network of Cluster 1. (C)ECG target -ALD pathogenic gene - PPI network of Cluster 2.

**
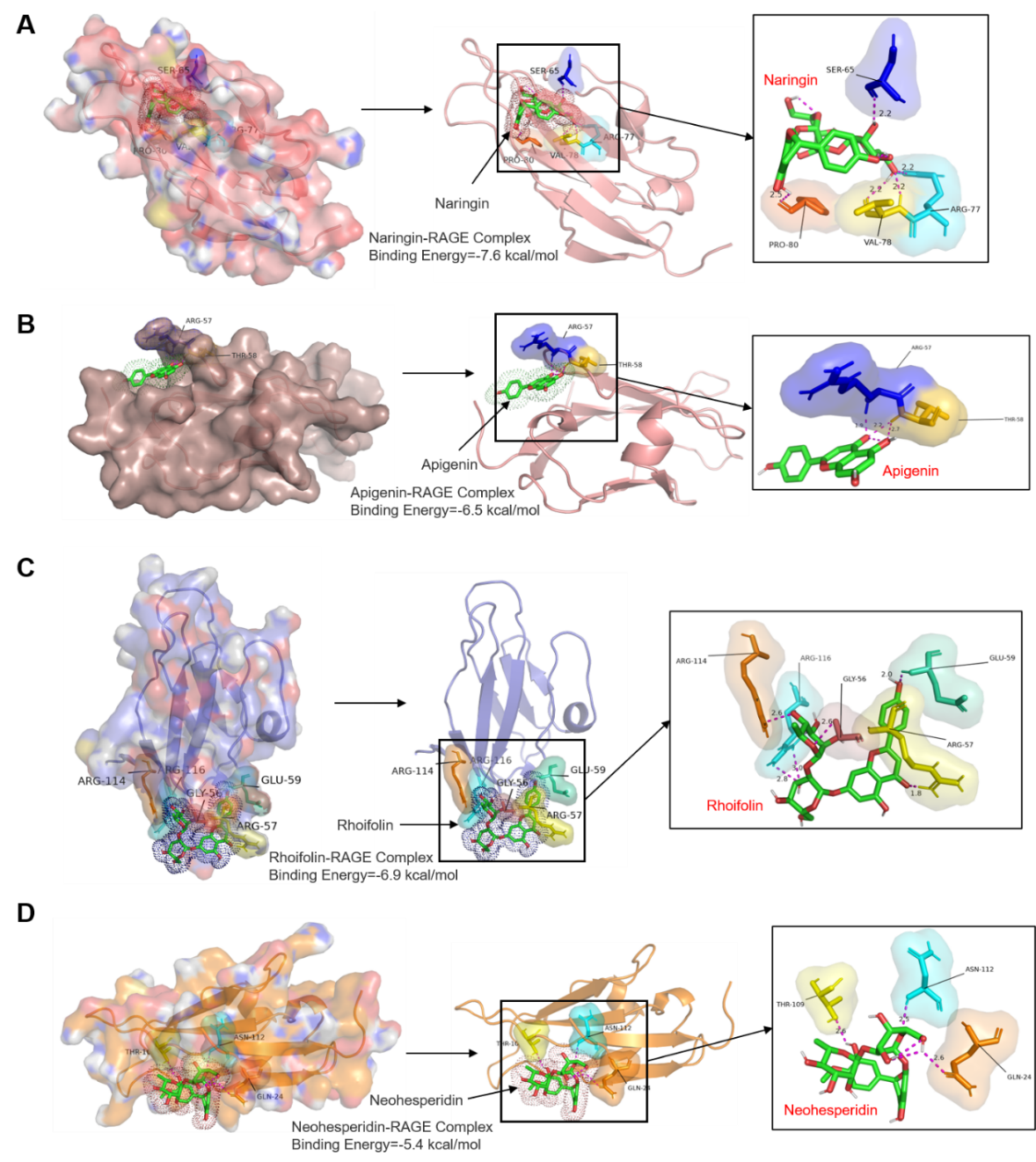
**

**FiguresS3.ECG extracts interacted with RAGE protein.** (A-D) The 3D pharmacophores of naringin-RAGE complex, apigenin-RAGE complex, rhoifolin-RAGE complex, neohesperidin-RAGE complex respectively. RAGE protein is shown in solid ribbon representation bound with naringin, apigenin, rhoifolin and neohesperidin respectively (green color).
